# Children With Inflammatory Bowel Disease in the COVID-19 Main Endemic Focus: The Lombardy Experience

**DOI:** 10.3389/fped.2021.607285

**Published:** 2021-04-21

**Authors:** Naire Sansotta, Lorenzo Norsa, Giovanna Zuin, Roberto Panceri, Dario Dilillo, Elena Pozzi, Costantino De Giacomo, Chiara Moretti, Rosaria Celano, Federica Nuti, Paola Sgaramella, Marina Di Stefano, Silvia Salvatore, Serena Arrigo, Valentina Motta, Lorenzo D'Antiga

**Affiliations:** ^1^Paediatric Hepatology Gastroenterology and Transplantation, Papa Giovanni XXIII Hospital, Bergamo, Italy; ^2^Department of Pediatrics, University of Milano Bicocca, FMBBM, San Gerardo Hospital, Monza, Italy; ^3^Department of Pediatrics, V. Buzzi Children's Hospital, University of Milan, Milan, Italy; ^4^Department of Pediatrics ASST Grande Ospedale Metropolitano Niguarda, Milan, Italy; ^5^Intermediate Pediatric Care Unit, Fondazione IRCCS Ca' Granda, Ospedale Maggiore Policlinico, Milan, Italy; ^6^Department of Pediatrics, IRCCS San Raffaele Scientific Institute, Milan, Italy; ^7^Department of Pediatrics, Ospedale “F. Del Ponte”, University of Insubria, Varese, Italy; ^8^Department of Pediatrics, Ospedale Maggiore, Crema, Italy

**Keywords:** inflammatory bowel disease, children, COVID-19, immunosuppression, SARS-CoV-2

## Abstract

**Objectives:** In the era of Coronavirus 2019 (COVID-19), concern has been raised for immunosuppressed patients, including children with inflammatory bowel diseases (IBD). We aimed to collect data from IBD tertiary centers of Lombardy during pandemic.

**Methods:** A cross-sectional survey enrolling IBD children has been completed by seven major IBD centers in Lombardy during lockdown. The clinical form included questions on any symptom consistent with severe acute respiratory syndrome coronavirus 2 (SARS-CoV-2) infection and the IBD adherence treatment. Furthermore, we have reviewed all IBD medical records including new IBD diagnoses and flares in known IBD patients after the lockdown.

**Results:** Questionnaires of 290 IBD children were returned during lockdown. Out of them, 24 children (8%) complained of mild symptoms suspicious of SARS-CoV-2 infection without needing hospitalization or changing IBD treatment. During the lockdown, one patient presented with IBD flare and one had infectious colitis, with no new IBD cases. Conversely, after lockdown, 12/290 (4%) children relapsed and 15 children were newly diagnosed with IBD. Last year, in the same timeframe, 20/300 (7%) children presented with IBD flare, while 17 children had IBD onset with no statistical difference.

**Conclusions:** Our data on children with IBD in a high COVID-19 prevalence region are reassuring. Only a minority of IBD children had mild symptoms, and no hospitalization or treatment modification was needed. Standard IBD treatments including biologics were safely continued. New IBD diagnoses and flares in known IBD children occurred after the lockdown phase, although no significant difference was found compared with the previous year.

## Introduction

An outbreak of a novel coronavirus [severe acute respiratory syndrome coronavirus 2 (SARS-CoV-2)] began in Wuhan, China (1, 2) in December 2019, and it has rapidly spread, with 100,362,196 confirmed cases of virus-related disease [Coronavirus 2019 (COVID-19)] worldwide and 2,151,794 deaths as of January 26, 2021 (https://www.worldometers.info/coronavirus/).

COVID-19 is a disease caused by the zoonotic coronavirus SARS-CoV-2 and transmitted from person to person through airborne droplets (3). A significant viral shedding has been described also in stools, raising the potential for a fecal–oral transmission and intestinal involvement (4).

In adults, COVID-19 predominantly presents with fever, chills, cough, shortness of breath, generalized myalgia, malaise, drowsiness, anosmia, hyposmia, and dysgeusia. A proportion of patients (4–20%) develop the most threatening complication represented by acute respiratory distress syndrome (5–7).

SARS-CoV-2 infection may also cause gastrointestinal symptoms such as vomiting and diarrhea (8). In a recent study, among 651 patients of the Zhejiang province, 11.4% presented with at least one gastrointestinal symptom and 10.8% had preexisting liver disease (9). Remarkably, the disease course of COVID-19 in children is predominantly benign, with mild or even no symptoms, and extremely rare fatalities (10, 11).

Following the outbreak in China, Italy was the first affected country in Europe, with the outbreak estimated to start on February 18. In particular, the Lombardy region became one of the areas with the highest prevalence of cases x 100,000 worldwide (https://www.bbc.com/news/world-europe-51645902). To date, Italy has reported 241,000 positive cases (confirmed with nasopharyngeal swabs for SARS-CoV-2), of whom 94,108 (39%) were in Lombardy (http://www.salute.gov.it/portale/nuovocoronavirus/), where 1/6 of the Italian population lives. However, it is estimated that at least 10% of the entire population (corresponding to 1 million out of 10 million people in the Lombardy region) has been exposed to the virus (http://spiral.imperial.ac.uk/handle/10044/1/77731).

Inflammatory bowel disease (IBD) is one of the most devastating and debilitating chronic gastrointestinal disorders that may affect children and adolescents (12). During SARS-CoV-2 outbreak, concern has been raised for the use of immunosuppressive treatments in patients with IBD (13).

The aim of our study was to collect all available data on pediatric IBD (PIBD) and SARS-CoV-2 exposure in the Lombardy region, in order to evaluate the impact of this pandemic on the management of PIBD patients. We investigated the presence of any symptom suspicious of SARS-CoV-2 reported by PIBD patients in addition to the IBD treatment during the pandemic. Furthermore, we analyzed the proportion of new IBD cases and flares that occurred in this time frame compared with those in the previous year.

## Materials and Methods

### Study Population

The study population included all PIBD patients followed in seven main PIBD centers in the Lombardy region. Demographic and clinical data were retrieved from the patients' medical charts. Disease activity was calculated at the time of the last available clinical follow-up using pediatric Crohn's disease (CD) and ulcerative colitis (UC) activity indexes: PCDAI and PUCAI. We have reviewed all electronic clinical charts of patients referred to our PIBD centers. All new diagnoses and IBD flare from May 5 to June 30 were identified, to seek the impact of reduced hospital attendance for follow-up visits during the lockdown. A comparison of the rate of new IBD diagnosis or relapses was made with the same period of the previous year.

### The Survey

A cross-sectional survey has been constructed in order to collect information on events that occurred during the lockdown period from February 21 to May 4. The questionnaire was given to all patients coming to the hospital for the scheduled infusion, while clinicians interviewed all other patients by telephone or email. Given the observational, retrospective nature of the study, a waiver was given on the need of approval by an ethics committee. However, verbal informed consent was obtained from all participants or the legally authorized representative at the time of the interview. The clinical form was based on questions regarding the occurrence of symptoms suspicious of COVID-19, in addition to the adherence to the current therapy. Furthermore, close contact with persons with COVID-19 in the previous weeks was also recorded. A “close contact” was defined by the Circular of the Italian Ministry of Health in any of the following situations: a person living in the same house of a COVID-19 case, who has had direct physical contact with a COVID-19 case (e.g., handshake), who has had direct (face-to-face) contact with a case of COVID-19, at <2 m and lasting longer than 15 min, who has been in a closed environment (e.g., classroom, meeting room, and hospital waiting room) with a case of COVID-19 in the absence of suitable personal protective equipment (PPE), who has traveled seated on an aircraft in the two adjacent seats, of a COVID-19 case, as well as his or her traveling companions or caregivers and crew members sitting in the section of the aircraft where the index case was seated in the previous 15 days (http://www.salute.gov.it/portale/nuovocoronavirus/dettaglioFaqNuovoCoronavirus.jsp?lingua=english&id=230). Based on clinical features, patients were defined as *highly suspected* for SARS-CoV-2 infection in the presence of at least one of the main respiratory symptoms: cough, fever, chest pain, difficult breathing, or *lowly suspected* in the presence of one of the additional symptoms: gastrointestinal symptoms (diarrhea, vomiting), fatigue, myalgia. In children who reported only diarrhea, clinical evaluation associated with blood and stool tests were performed to discriminate PIBD flares from gastrointestinal infections or COVID-19. Endoscopy was reserved for an emergency situation during the lockdown period.

### Diagnostic Tests

Nasopharyngeal swab availability was limited and performed only in the case of severe symptoms leading to hospital admission, before elective procedures (e.g., upper and lower endoscopy) or, in some cases, before biologic infusions in day hospital setting, according to the regional health care system-established recommendations (https://www.cdc.gov/coronavirus/2019-ncov/symptoms-testing/index.html). Serological tests have been introduced only recently, but are still not easily available for asymptomatic children.

### Statistical Analysis

Statistical analysis was performed using IBM SPSS Statistics 21. Categorical data were summarized in absolute frequencies and percentages. The Kolmogorov–Smirnov test was used to assess Gaussian distribution of the data. Quantitative variables were summarized with mean ± standard deviation or median and range when appropriate. The comparison of the frequencies of the categorical variables between groups was analyzed by Chi-square test or Fisher's Exact test. Statistical significance was defined at *p* < 0.05.

## Results

### Pediatric Inflammatory Bowel Disease Patients and COVID-19 Symptoms

During the lockdown, out of 308 PIBD children regularly followed in seven centers in our region, 290 completed the survey, with a response rate of 94%. The median age of the respondents was 15.2 years (range 2–18 years); 52% were male. The study population comprised 117 (40%) patients diagnosed with CD, 155 (54%) with UC, and 18 (6%) with IBD-unclassified (IBD-U). As far as treatment, in our cohort, 96/290 patients (33%) were exclusively on salicylates, while 87/290 (30%) were on biologic therapy. Among those on biologics, 44/87 (50%) were on infliximab (monotherapy in 40), 43/87 (49.5%) were on adalimumab (monotherapy in 36), while two patients were on ustekinumab, and Vedolizumab, respectively. Approximately a third of our study population was on other immunosuppressive treatments such as thiopurines, steroids, and thalidomide. The demographic and clinical features of our study population are described in Table 1. Between February 21 and May 4, 24 PIBD patients (8%) complained of symptoms consistent with SARS CoV-2 infection. Out of them, 22 (92%) reported mainly with respiratory symptoms and were defined highly suspected for COVID-19, while two experienced minor symptoms and were defined as lowly suspected (Table 2). The most common symptom was fever in 20 children (80%) followed by cough (28%), fatigue (8%), and vomiting (4%). Diarrhea was reported only in two children. No child complained of hyposmia or dysgeusia. All patients recovered without requiring hospitalization. Among the symptomatic patients, 10/24 (42%) were on thiopurines, followed by 7/24 (30%) on salicylates, 4/24 (16%) on biologic treatment, and 3/24 (12%) off therapy.

**Table 1:** Demographic and clinical features of the study population during the pandemic.

Number of patients	290
Female (%)	138 (48%)
Age, median (range)	15.2 (2–18)
Disease phenotype:	
• Crohn's disease (%)	117 (40%)
• Ulcerative colitis (%)	155 (54%)
• IBD U	18 (6%)
Previous IBD-related surgery (%)	11 (4%)
Comorbidity (obesity, liver disease, other autoimmune disorders, genetic syndromes)	21 (7%)
Treatments:	
• Anti-inflammatory (salicylates) (%)	96 (33%)
• Thiopurines or methotrexate (%)	72 (25%)
• Biologics (infliximab, adalimumab, ustekinumab, vedolizumab) (%)	87 (30%)
• Thalidomide (%)	8 (3%)
• Steroids (%)	8 (3%)
• Other therapies (exclusion diet, probiotics) (%)	6 (2%)
• Off therapy (%)	13 (4%)

*IBD-U, inflammatory bowel disease unclassified*.

**Table 2:** Clinical features of likely pediatric inflammatory bowel disease (PIBD) cases presented with suspected severe acute respiratory syndrome coronavirus-2 (SARS-CoV-2) symptoms.

**Patient**	**Disease**	**Paris classification**	**Treatment**	**Disease activity**	**Symptoms**	**COVID-19 contact/nasopharyngeal swab**
1	UC	E4	5-ASA	Remission	Cough	No/not done
2	CD	L3	ADA	Remission	Cough	No/not done
3	CD	L3	AZA	Remission	Fever	No/not done
4	CD	L3 + L4a	AZA	Remission	Myalgia, fatigue	Yes/not done
5	CD	L3	AZA	Remission	Fever	No/not done
6	CD	L2	AZA	Remission	Fever	No/not done
7	CD	L2	AZA	Remission	Fever	No/not done
8	UC	E4	5-ASA	Remission	Fever	No/not done
9	UC	E4	AZA	Mild	Fever	Yes/negative
10	UC	E2	5-ASA	Remission	Diarrhea	No/not done
11	UC	E2	AZA	Remission	Fever	No/not done
12	UC	E2	5-ASA	Remission	Fever	No/not done
13	CD	L3	IFX	Remission	Fever, fatigue	*Yes/positive*
14	UC	E2	5-ASA	Mild	Cough	No/not done
15	CD	L1	AZA	Remission	Fever, cough	No/not done
16	IBD-U	E1	Off-therapy	Remission	Fever	No/not done
17	UC	E4	Off-therapy	Remission	Fever, cough	No/not done
18	CD	L3	AZA	Remission	Fever, vomiting, diarrhea	No/not done
19	UC	E4	5-ASA	Remission	Fever	No/not done
20	UC	E4	5-ASA	Remission	Fever	No/not done
21	CD	L3	IFX	Remission	Fever, rhinitis	No/not done
22	CD	L3	AZA	Remission	Fever, cough	No/not done
23	CD	L1	CDED	Remission	Fever, fatigue	No/not done
24	UC	E4	IFX	Remission	Fever, cough, rhinitis	No/not done

*ADA, adalimumab; 5-ASA, salicylates; AZA, azathioprine, CD, Crohn's disease; CED, Crohn disease exclusion diet; IFX, infliximab; UC, ulcerative colitis*.

Risk factors such as obesity and liver disease (sclerosing cholangitis and autoimmune hepatitis) were identified in three symptomatic patients (11%). Of interest, 10/290 children were reported to be in close contact with persons confirmed with COVID 19 infection, and out of them, four (40%) presented with suspicious symptoms.

### Pediatric Inflammatory Bowel Disease New Cases and Flares

During the lockdown, no new IBD cases were reported, while one patient presented with IBD flare and another one had infectious colitis. Of note, both of them (0.7% of our study population) required hospital admission due to fever and gastrointestinal symptoms. A 16-year-old male with CD on thiopurines presented with a 14-day history of diarrhea and vomiting. At admission to the hospital, he was tested for SARS-CoV-2, which resulted negative. He was eventually diagnosed with IBD flare and was therefore switched to infliximab with subsequent achievement of remission. The other one was a 16 year-old female with UC on infliximab and azathioprine combination therapy, who complained of fever and bloody diarrhea. Her nasopharyngeal swab, performed because of hospitalization, yielded a negative result. A stool culture was positive for *Salmonella* species.

The survey revealed that all our IBD children were adherent to therapy. Forty had their scheduled infusions (infliximab and vedolizumab) during the study period, and none of them delayed or discontinued the treatment because of the epidemic. Following the lockdown phase (from May 5 to June 30), we identified 15 new PIBD diagnoses and 12 flares in known IBD cases. In 9/15 (60%) children newly diagnosed with IBD, the onset of gastrointestinal symptoms occurred during the lockdown period, and the delay of referral was due to the COVID-19 fear. All of them had a nasopharyngeal swab test for SARS-CoV-2 infection because of elective endoscopy, and they were found to be negative (Table 3A). Conversely, all patients reporting an IBD flare presented with symptoms starting after the lockdown phase. Out of 12 children with PIBD flare, five (42%) needed to change their immunosuppressive therapy. Most of them (8/12, 66%) were tested for SARS-CoV-2 by nasopharyngeal swab that was negative in 8/8 children (Table 3B). Of interest, during the previous year, from February to June 2019, 17 children had IBD onset, while 20/300 presented with IBD flare, with no statistical difference from the current year (respectively, *p* = 0.28, *p* = 0.44).

**Table 3A:** Clinical features of new PIBD patients and SARS-CoV2 diagnostic testing.

**Patient**	**Disease**	**Paris classification**	**Induction treatment**	**SARS-CoV2 nasopharyngeal swab**
1	VEO-UC	E4	PDN-IFX	Negative
2	CD	L3	EEN-AZA	Negative
3	CD	L3	EEN-AZA	Negative
4	VEO-CD	L3	PDN	Negative
5	UC	E3	PDN/5-ASA	Negative
6	CD	L2	PDN/IFX	Negative
7	CD	L3,L4a	IFX	Negative
8	UC	E4	PDN/IFX	Negative
9	UC	E4	5-ASA	Negative
10	CD	L3	PEN/AZA	Negative
11	UC	E4	5-ASA	Negative
12	CD	L3	PEN/AZA	Negative
13	UC	E4	5-ASA	Negative
14	CD	L1	CDED	Negative
15	Cd	L3	CDED-IFX,AZA	Negative

*5-ASA, salicylates; AZA, azathioprine; CD, Crohn's disease; CDED, Crohn disease exclusion diet; EEN, exclusive enteral nutrition; IFX, infliximab; PDN, prednisone; PEN, partial enteral nutrition; VEO, very early onset; UC, ulcerative colitis*.

**Table 3B:** Clinical features of PIBD flares and SARS-CoV2 diagnostic testing.

**Age, gender**	**Disease**	**Paris classification**	**Previous treatment**	**Re-treatment**	**PUCAI/PCDAI**	**SARS-CoV2 nasopharyngeal swab**
1	UC	E4	AZA	BCL	Flare: PUCAI 50	Not available
2	CD	L1 + L3	AZA	PDN-ADA	Flare: PCDAI 35	Negative
3	CD	L1 + L3	ADA	PDN-UST	Flare: PCDAI 45	Negative
4	CD	L3	AZA	BDN	Flare: PCDAI 50	Negative
5	UC	E4	AZA	IFX	Flare: PUCAI 45	Not available
6	UC	E4	AZA	PDN	Flare: PCDAI 35	Negative
7	UC	E4	ASA	PDN	Flare: PUCAI 30	Negative
8	UC	E4	AZA	PDN-IFX	Flare: PUCAI 30	Not available
9	UC	E4	AZA-ADA	PDN-VEDO	Flare: PUCAI 30	Negative
10	UC	E4	ASA	BCL	Flare: PUCAI 25	Not available
11	CD	L3	ADA	PDN-THALI	Flare: PCDAI 45	Negative
12	CD	L3,L4a	ADA	IFX	Flare: PCDAI 35	Negative

*AZA, azathioprine; CD, Crohn's disease; IFX, infliximab; PCDAI, Pediatric Crohn Disease Activity Index; PDN, prednisone; PUCAI, Pediatric Ulcerative Colitis Activity Index; THALI, thalidomide; VEDO, vedolizumab; UC, ulcerative colitis*.

### Nasopharyngeal Swab Test and Serologic Testing

Among 290 PIBD children who completed the survey, 25 (9%) had nasopharyngeal swab for SARS-CoV-2 done for the following reasons: 12/25 in-hospital biologic infusion, 8/25 IBD flare, 3/25 ascertained contact with a confirmed COVID-19 case, and 2/25 hospital admission. Two patients had positive nasopharyngeal swab test. Both of them had close contacts with a confirmed COVID-19 case, and only one was symptomatic, complaining of fatigue. In addition, all 15 children newly diagnosed with IBD were tested and had negative nasopharyngeal swabs. Serologic tests were performed in 12/290 patients (4%), and two of them (17%) had evidence of SARS-CoV2 antibodies, with negative nasopharyngeal swab and without evidence of previously reported suggestive symptoms. No newly diagnosed IBD children had serological test done.

## Discussion

The current survey was conducted among seven PIBD centers of Lombardy, which is, to date, the highest epidemic focus in Italy and one of the main epicenters of the epidemic in Europe. In order to estimate the prevalence of symptoms potentially related to SARS-CoV-2, a very large population of IBD children was interviewed. To the best of our knowledge, few pediatric data have been published so far on children with IBD during the pandemic.

In the 11 weeks of lockdown period, up to May 4, in our PIBD Centers, 24/290 PIBD patients (8%) complained of some symptoms possibly related with SARS-CoV-2 infection, and 22 out of 24 children were defined as highly suspected of COVID-19. Despite that it can be hypothesized that children with IBD might be at increased risk of SARS-CoV-2 infection, and that the risk of a severe clinical course of COVID-19 might be increased in patients on immunomodulatory treatment, in our study population, no severe course of the condition was observed. Symptoms reported by all patients were mild (mostly fever and cough). The only previous report found eight IBD children with COVID-19 among 102 PIBD centers affiliated with the Porto and ESPGHAN groups; among them only six had nasopharyngeal–oropharyngeal confirmation. None required hospitalization because of SARS-CoV2-related infection (14). In addition, an International registry called SECURE IBD (Surveillance Epidemiology of Coronavirus Under Research Exclusion) has reported so far 209 children with confirmed COVID-19 infection. Children had a mild course; only 14 (7%) required hospitalization. Increasing age, comorbidities, and corticosteroids are found to be associated with severe COVID-19 among IBD patients, although a causal relationship cannot be definitively be established (15). On the other hand, immunosuppression and TNF antagonists do not appear to be associated with severe COVID-19 (16, 17).

Remarkably, none of our patients modified the current treatment regimen, consisting of salicylates, immunomodulators, or biologics not even during the lockdown phase. Furthermore, all children had their scheduled infusions (infliximab and vedolizumab) or injections during the lockdown period, and none delayed or withheld it because of the epidemic (17). Noteworthy, in China and South Korea, it has been reported that biologic treatment has been delayed in 79 children, of whom 17 (22%) had exacerbation of their IBD (14). Those data highlight the risk of modifying patients' regimens without scientific evidence supporting it, which may have a significant impact on the health of IBD patients (14). In addition, a case of a 14-year-old boy with recently diagnosed small bowel, perianal CD presented with 5 days of fever and abdominal pain has been described. Of interest, he was diagnosed with multisystem inflammatory syndrome (MIS) related to COVID-19 (SARS-CoV-2 PCR positive) and treated successfully with infliximab to manage both entities (18). Furthermore, pediatric patients with autoinflammatory diseases, including those receiving biologic treatment and/or colchicine, may not be at increased risk of infection and severe disease course (2).

In our study population, 21 IBD children had some comorbidity (obesity, liver disease, genetic syndrome, and autoimmune disorder) and three of them (14%) developed SARS-CoV2 symptoms without severe course. The majority of symptomatic children (92%) were older than 10 years.

Individuals living in or having visited places where community spread of SARS-CoV-2 is reported are at elevated risk of infection, and this increases with contact with persons known to be infected by SARS-CoV-2 (13). In our study, out of 24 symptomatic patients, four had close contacts with highly suspected or confirmed COVID-19 cases. Conversely, among 10 patients with ascertained contact with a confirmed COVID-19 case, four (40%) had some symptoms. Having said that, we cannot rule out an asymptomatic carrier status in the remaining patients or limited spreading of the virus due to social isolation during the lockdown.

In our cohort, we had two adolescents admitted to the hospital with fever and gastrointestinal symptoms during the lockdown. Nasopharyngeal testing was carried out, as per the local in-hospital screening protocol. Both of them were found to be negative. Of interest, they were eventually diagnosed with an IBD flare and a *Salmonella* infection, respectively. Even though some COVID-19 patients can present with gastrointestinal symptoms (19), this should not lead to an overlook of other possible causes, especially in IBD patients. For this reason, in this setting, it is extremely important to maintain a high index of suspicion for COVID-19, while continuing to carry out a complete differential diagnostic work up in agreement with PIBD guidelines.

Remarkably, during the lock down, we reported one IBD flare with no new IBD diagnoses. Following the lockdown phase, we identified 15 children newly diagnosed with IBD, while 12/290 (4%) known PIBD children presented with an IBD flare. Most of the newly diagnosed children with IBD had symptom onset during the pandemic, and two possible factors were responsible for the diagnostic delay. First, elective procedures (upper and lower gastrointestinal endoscopy) were put on hold through the pandemic, and most hospital referrals were delayed given the SARS-CoV-2 fear. Second, children with IBD flare complained of some symptoms nearly after the lockdown phase. The role of SARS-CoV-2 on these relapses is still uncertain because pharyngeal swabs were negative, but no one of them had a serological test carried out so far. Conversely, we might speculate that there was no significant delay in recognizing them during the pandemic.

In addition, the incidence rate of new IBD diagnoses and flares was found not statistically significant when compared with that of the previous year, in the same interval time.

The major limitation of our study is the lack of nasopharyngeal swab and serological testing in most patients, determined by the early period of assessment of these patients (before testing was widely available) and regional health care system indications (that included only patients with respiratory symptoms and fever), with likely underestimation of SARS-CoV-2 infection. Given that, we cannot assess the exact proportion of children that presented with SARS-CoV-2 infection during the pandemic, but recent studies suggest that, in highly endemic areas, the rate of contact with the virus is likely as high as 10% of the entire population, with a high proportion of exposed people who remained asymptomatic or with very mild symptoms (http://spiral.imperial.ac.uk/handle/10044/1/77731). In addition, it has been shown that 56.9% of people living in our city and 30.6% of health workers in our hospitals had positive serological testing (data available on http://www.ats-bg.it/upload/asl_bergamo/gestionedocumentale/CSATSBG2020-06-08coronavirusesitissierologici_784_31055.pdf). Taking into account these limitations, the aim of our study was not to quantify the rate of the contact of PIBD children with the virus but highlight that children with IBD do not have any increased risk of having severe course of SARS-CoV-2 infection in a highly endemic area.

## Conclusion

Our study, carried out at the epicenter of SARS-CoV-2 Italian outbreak, highlights that children, despite being affected by IBD and treated with immunosuppressive agents, may have mild clinical COVID-19 course, with no need for hospitalization and no severe complications. Standard IBD treatments including biologics were safely continued through the pandemic. Although some new IBD diagnoses and flares in known PIBD patients occurred shortly after the lockdown, their incidence rate was found not significantly different from the previous year.

Even though the lack of confirmation testing of SARS-CoV2, either nasopharyngeal swab test or serologic sample, represents an important limitation of our study, we might conclude that children with PIBD can be managed safely and effectively during the SARS-CoV-2 pandemic, and that in this group of patients, there is no need to modify the treatment strategies.

### Take Home Messages

- Children with IBD do not seem to have an additional risk for COVID-19 despite immunosuppressive therapy.
- Standard treatment for IBD can be safely continued to maintain the control of the disease during the pandemic.
- No change in IBD incidence and severity was reported during and after the pandemic.

## Data Availability Statement

The data analyzed in this study is subject to the following licenses/restrictions: database. Requests to access these datasets should be directed to naire.sansotta@gmail.com.

## Author Contributions

NS contributed to the study concept and design, acquisition of data, analysis and interpretation of data, and drafting of the manuscript. LN contributed to the study concept and design and critical revision of the manuscript. GZ, RP, DD, EP, CG, CM, RC, FN, PS, MS, SS, SA, and VM contributed to the acquisition of data and critical revision of the manuscript. LD'A contributed to the study concept and critical revision of the manuscript for important intellectual content. All authors contributed to the article and approved the submitted version.

## Conflict of Interest

The authors declare that the research was conducted in the absence of any commercial or financial relationships that could be construed as a potential conflict of interest.

## Abbreviations

CD: Crohn's disease
COVID 19: Coronavirus 2019 COVID-19
IBD: inflammatory bowel disease
IBD-U: IBD-unclassified
PIBD: Pediatric inflammatory bowel disease
SARS-CoV-2: severe acute respiratory syndrome coronavirus 2
UC: ulcerative colitis.

## References

[1] Sohrabi C Alsafi Z O'Neill N Khan M Kerwan A Al-Jabir A. World Health Organization declares global emergency: a review of the 2019 novel coronavirus (COVID-19). Int J Surg. (2020) 76:71–6. 10.1016/j.ijsu.2020.02.034 32112977 PMC7105032

[2] Haslak F Yildiz M Adrovic A Sahin S Koker O Aliyeva A. Management of childhood-onset autoinflammatory diseases during the COVID-19 pandemic. Rheumatol Int. (2020) 40:1423–31. 10.1007/s00296-020-04645-x 32661928 PMC7355083

[3] Zhou P Yang XL Wang XG Hu B Zhang L Zhang W. A pneumonia outbreak associated with a new coronavirus of probable bat origin. Nature. (2020) 579:270–3. 10.1038/s41586-020-2012-7 32015507 PMC7095418

[4] Xiao F Tang M Zheng X Liu Y Li X Shan H. Evidence for gastrointestinal infection of SARS-CoV-2. Gastroenterology. (2020) 158:1831.e3–3.e3. 10.1053/j.gastro.2020.02.055 32142773 PMC7130181

[5] Deng Y Liu W Liu K Fang YY Shang J Zhou L. Clinical characteristics of fatal and recovered cases of coronavirus disease 2019 in Wuhan, China: a retrospective study. Chin Med J (Engl). (2020) 133:1261–7. 10.1097/CM9.0000000000000824 32209890 PMC7289311

[6] Yang W Cao Q Qin L Wang X Cheng Z Pan A. Clinical characteristics and imaging manifestations of the 2019 novel coronavirus disease (COVID-19): a multi-center study in Wenzhou city, Zhejiang, China. J Infect. (2020) 80:388–93. 10.1016/j.jinf.2020.02.016 32112884 PMC7102539

[7] Guan WJ Ni ZY Hu Y Liang W Ou C He L. Clinical characteristics of coronavirus disease 2019 in China. N Engl J Med. (2020) 382:1708–20. 10.1056/NEJMoa2002032 32109013 PMC7092819

[8] Gu J Han B Wang J. COVID-19: gastrointestinal manifestations and potential fecal-oral transmission. Gastroenterology. (2020) 158:1518–9. 10.1053/j.gastro.2020.02.054 32142785 PMC7130192

[9] Jin X Lian JS Hu JH Gao J Zheng L Zhang YM. Epidemiological, clinical and virological characteristics of 74 cases of coronavirus-infected disease 2019 (COVID-19) with gastrointestinal symptoms. Gut. (2020) 69:1002–9. 10.1136/gutjnl-2020-320926 32213556 PMC7133387

[10] Sun D Li H Lu XX Xiao H Ren J Zhang FR. Clinical features of severe pediatric patients with coronavirus disease 2019 in Wuhan: a single center's observational study. World J Pediatr. (2020) 16:251–9. 10.1007/s12519-020-00354-4 32193831 PMC7091225

[11] Lu X Zhang L Du H Zhang J Li YY Qu J. SARS-CoV-2 infection in children. N Engl J Med. (2020) 382:1663–5. 10.1056/NEJMc2005073 32187458 PMC7121177

[12] Roberts SE Thorne K Thapar N Broekaert I Benninga MA Dolinsek J. A systematic review and meta-analysis of paediatric inflammatory bowel disease incidence and prevalence across Europe. J Crohns Colitis. (2020) 14:1119–48. 10.1093/ecco-jcc/jjaa037 32115645

[13] Mao R Liang J Shen J Ghosh S Zhu LR Yang H. Implications of COVID-19 for patients with pre-existing digestive diseases. Lancet Gastroenterol Hepatol. (2020) 5:425–7. 10.1016/S2468-1253(20)30076-5 32171057 PMC7103943

[14] Turner D Huang Y Martin-de-Carpi J Aloi M Focht G Kang B. Corona virus disease 2019 and paediatric inflammatory bowel diseases: global experience and provisional guidance (March 2020) from the Paediatric IBD Porto Group of European Society of Paediatric Gastroenterology, Hepatology, and Nutrition. J Pediatr Gastroenterol Nutr. (2020) 70:727–33. 10.1097/MPG.0000000000002729 32443020 PMC7273950

[15] Brenner EJ Pigneur B Focht G Zhang X Ungaro RC Colombel JF. Benign evolution of SARS-Cov2 infections in children with inflammatory bowel disease: results from two international databases. Clin Gastroenterol Hepatol. (2021) 19:394.e5–6.e5. 10.1016/j.cgh.2020.10.010 33059040 PMC7550063

[16] Brenner EJ Ungaro RC Gearry RB Zhang X Ungaro RC Colombel JF. Corticosteroids, but not TNF antagonists, are associated with adverse COVID-19 outcomes in patients with inflammatory bowel diseases: results from an international registry. Gastroenterology. (2020) 159:481.e3–91.e3. 10.1053/j.gastro.2020.07.020 32425234 PMC7233252

[17] D'Antiga L. Coronaviruses and immunosuppressed patients: the facts during the third epidemic. Liver Transpl. (2020) 26:832–4. 10.1002/lt.25756 32196933

[18] Dolinger MT Person H Smith R Jarchin L Pittman N Dubinsky MC. Pediatric Crohn disease and Multisystem Inflammatory Syndrome in Children (MIS-C) and COVID-19 treated with infliximab. J Pediatr Gastroenterol Nutr. (2020) 71:153–5. 10.1097/MPG.0000000000002809 32452979 PMC7268863

[19] Luo S Zhang X Xu H. Don't overlook digestive symptoms in patients with 2019 novel coronavirus disease (COVID-19). Clin Gastroenterol Hepatol. (2020) 18:1636–7. 10.1016/j.cgh.2020.03.043 32205220 PMC7154217

